# Fuel subsidy reform and the social contract in Nigeria: A micro-economic analysis^☆^

**DOI:** 10.1016/j.enpol.2021.112336

**Published:** 2021-09

**Authors:** Neil McCulloch, Tom Moerenhout, Joonseok Yang

**Affiliations:** a The Policy Practice and an Associate Fellow at the Institute of Development Studies, Brighton, UK, Address: 67 Bicester Road, Kidlington, Oxfordshire, OX5 2LD, UK; b Columbia University and Senior Associate at the International Institute for Sustainable Development, Address: 420 W 118th Street # 1410, New York, NY, 10027, USA; c Sungkyunkwan University, 25-2 Sungkyunkwan-ro, Myeongnyun 3(sam)ga-dong, Jongno-gu, Seoul, 03063, South Korea

**Keywords:** Fuel subsidy reform, Political economy, Perceptions, Corruption, Nigeria, Social contract

## Abstract

Fuel subsidies in Nigeria are enormous – around USD 3.9 billion – almost double the health budget. Such subsidies come at great cost: the opportunity costs of such spending on other development objectives are large; the distribution of resources to the state governments is reduced; the vast majority of the subsidy goes to better off Nigerians; and cheaper petrol encourages greater pollution, congestion and climate change. Despite this, most Nigerians oppose the reduction of subsidies. We draw on a new nationally representative household survey that asked Nigerian men and women about their knowledge and attitudes towards subsidies. We construct and test a set of hypotheses about the factors associated with support for subsidy reform. We find that those who pay more or who experience less availability of fuel tend to support reform more. On the other hand, people who believe the Government is corrupt or lacks the capacity to implement compensation programs appear strongly opposed to reform. Finally, being religious and the delivery of reasonable national and local services also improves the acceptance of reform. These results support the idea that building a social contract is key to reform success.

## Highlights

• Results from a new household survey in Nigeria (N = 16,000) on subsidy reforms.
• Economic factors and service delivery improve acceptance of gasoline subsidy reform.
• Beliefs about corruption are strongly correlated with opposition to subsidy reform.
• Beliefs about incompetence to use subsidy savings also correlate with opposition.
• Results support the idea that building a social contract is key to reform success.

## 1 Introduction

Fossil fuel subsidies are large and widespread. The International Energy Agency estimated the value of fossil fuel consumption subsidies^1^ globally as $325 billion in 2015, up to more than $400 billion in 2018 (International Energy Agency, 2019). This is significantly larger than the value of all aid ($163 billion in 2015) – McCulloch (2017) shows that, for the 96 countries where there is data for both energy subsidies and aid, 59 per cent have subsidies that are larger than all the bilateral aid that they receive. Energy subsidies are particularly prevalent in the Middle East and Africa – even in sub-Saharan Africa, the median country has energy subsidies of over 1 per cent of GDP (McCulloch and Dom, 2019) – making subsidies larger than some important sources of domestic revenue in several countries. Hoy and Sumner (2016) calculate that fossil fuel subsidies amounted to 69% of the global poverty gap in 2011.^2^ Moreover, this only takes into account the fiscal cost of subsidies. When including the cost of negative externalities induced by such subsidies – such as air pollution, greenhouse gas emissions, congestion, accidents, road damage, and foregone tax revenue – the IMF estimate of the cost of energy subsidies rises to $5.3 trillion, or over 6 per cent of global GDP (Coady et al., 2015).

There is extensive literature on the harm caused by such subsidies. Fossil fuel subsidies typically encourage greater consumption of such fuels leading to higher levels of air pollution. Outdoor air pollution from fossil fuels as well as other sources was responsible for an estimated 3.2 million premature deaths a year worldwide in 2012 (Coady et al., 2015). Fossil fuel subsidies also encourage greater greenhouse gas emissions. The IMF estimates that CO2 emissions would be reduced by 24 per cent if prices were to be increased to their efficient level (Coady et al., 2015), although the size of this impact has been challenged recently by Jewell et al. (2018). Estimates also exist for the additional congestion, accidents, and road damage caused by fossil fuel subsidies (van Benthem, Arthur, 2015). Moreover, fossil fuel subsidies are inequitable; Arze del Granado, Coady, and Gillingham (2012) estimated the welfare impact for 20 countries in Africa, Asia, the Middle East, and Latin America. They found that fuel subsidies are an extremely costly approach to helping the poor, with the top income quintile typically capturing six times more in subsidies than the bottom.

Given the harmful nature of fossil fuel subsidies, it is perhaps surprising that there has not been more concerted effort to remove them.^3^ In general, progress on fossil fuel subsidy reform has been mixed, at best (Ross et al., 2017). The answer lies in the complex political economy of subsidy reform (Victor, 2009; van Asselt and Skovgaard, 2018; Inchauste and Victor, 2017). Politicians in all countries are reluctant to implement reforms, particularly where they may result in a significant increase in fuel or electricity prices. This is especially the case in countries where no effective mechanisms exist for protecting the poor or compensating those worst affected (Moerenhout, 2017). Subsidy mechanisms are also notoriously prone to corruption; this creates vested interests who strongly oppose reform (Inchauste et al., 2018; Coxhead and Grainger, 2018), as well as popular political movements that use reform episodes as an opportunity to protest against governments that are felt to be illegitimate (Hossain et al., 2018).

Given the overwhelming evidence that the removal of fossil fuel subsidies would be beneficial if the resources were deployed to more developmental purposes, how can governments that wish to pursue reform persuade their populations to support, or at least accept such changes? Recent evidence suggests that communication is a key part of successful reform efforts – governments that have made clear the reasons for reform, compensated those worst affected, and ensured that the benefits are widely shared have tended to be more successful (Kojima, 2016; Rentschler and Bazilian, 2017). However, there is, to date, relatively little evidence about which factors make people more likely to support reform.

This paper provides rigorous empirical evidence about the determinants of support for fuel subsidy reforms in Africa's largest economy – Nigeria. Fuel subsidies in Nigeria are large – at the last estimate, the state subsidizes petrol to the tune of around USD 3.9 billion (Munshi, 2018) – almost double the government's spending on health.^4^ Subsidies exist because the government fixes the price of petrol for consumers below the international price, and uses government resources to pay for the difference.^5^ Despite numerous attempts at reform, Nigeria has never successfully removed petrol subsidies,^6^ in large part because of strong popular opposition to reform; Nwachukwu et al. (2013) as well as Akov (2015) have highlighted the large level of petrol subsidies and the coalition of interest groups that have cooperated to protect the continuation of subsidization.

We draw on a new large, nationally representative dataset of the perceptions of Nigerians regarding taxes and subsidies (McCulloch and Moerenhout, 2019) to assess the factors that make people more or less likely to support reform. Support for reform is low, at under 30 per cent of the population. However, we find that economic variables (related to the price and availability of fuel), along with better service delivery by government, are associated with greater support for reform, while negative views of corruption and a lack of capacity in the government are associated with opposition to reform.

Our paper is structured as follows. The next section reviews the existing literature on the perceptions of subsidies and offers some hypotheses about the determinants of support for reform. The following section describes our data, the variables used, and the model we employ. Our results are then presented and discussed. The final section concludes with some lessons for policy and further research.

## 2 Literature and hypotheses

Our theoretical framework draws upon the literature on perceptions about tax compliance and tax morale (Luttmer and Singhal, 2014; McCulloch et al., 2019). In particular, we explore six potential factors that may influence support for subsidy reform.

As in the literature on tax compliance (Allingham and Sandmo, 1972), we would expect economic factors to have an influence on support for subsidy reform. Since subsidy reform generally entails an increase in fuel prices, we would expect those with high consumption to be more opposed to reform. On the other hand, those experiencing high prices when paying for fuel (suggesting they do not receive the subsidy) may be more in favour of reform. Availability may also influence support for reform. For electricity, Sharma et al. (2018) found that households in Uttar Pradesh, India, are more willing to accept tariff increases if the availability of supply improves, and Garg et al. (2016) found that availability and reliability have a positive impact on accepting reform. Similarly, consumers may support reform if it improves the availability of fuel (Alkon et al., 2016).

Trust in government, in general, appears to influence peoples' openness to subsidy reforms (Moerenhout et al., 2017; Inchauste and Victor, 2017). Government credibility is linked to a perception of the government's ability to implement reforms and redistribute or reinvest savings from reforms (Beaton et al., 2013; Bridel and Lontoh, 2014; Baig et al., 2007; Indriyanto et al., 2013; Scobie, 2018). In the Nigerian context, some authors have suggested that there is a trust deficit (Ogbu, 2012), with many reform opponents such as labour unions and civil right groups highlighting the inability of governments to protect the poor (Soile and Mu, 2015; Bashir, 2013; Nwachukwu et al., 2013; Africa Research Bulletin, 2011; Akov, 2015).

In many countries, including Nigeria, fuel subsidies are regarded as part of an implicit social contract between citizens and the state (Beblawi and Luciani, 1987; Hertog, 2017; Luciani, 1990; Fattouh et al., 2016; Moerenhout et al., 2017). Therefore, reducing subsidies without improving social welfare protection may be considered a unilateral change in the social contract, which can give rise to protest and political instability (Moerenhout, 2018a, b; Luciani, 1990). Conversely, if people believe that the state is fulfilling its obligations by providing better services and transparently investing the savings from fuel subsidy reform into sectors of direct relevance to households (e.g., health, education, and infrastructure), then they may be willing to accept subsidy reform, in the same way as improved services can increase support for tax compliance (Bodea and LeBas, 2016; McCulloch et al., 2019).^7^

There is a large literature on the effect of social and personal norms on tax compliance and tax morale (Jimenez and Iyer, 2016) as well as the influence of societal norms more indirectly (Bobek et al., 2013; Bobek et al., 2007; Damayanti and Baridwan, 2015). There is also literature on the impact of religion and religiosity on tax compliance and tax morale (Torgler et al., 2008; Richardson, 2008). However, tax payment is typically a choice, whereas whether one receives a fuel subsidy is not. As a result, there is very little literature on the connection between personal and social norms and support for subsidy reform. There is some evidence that citizens from resource-rich countries believe that resource wealth should be distributed among the population, including by lowering the prices of the resource (Chelminski, 2018). In some OPEC countries, there remains a belief that certain sections of society deserve some form of subsidized energy (Hochman and Zilberman, 2015), and the same argument has been made in the Nigerian context (Nwachukwu et al., 2013). Furthermore, support for pricing reform is clearly influenced by the extent to which those in the same community support reform (Blankenship, Wong, and Urpelainen, 2019).

In countries all over the world, household surveys show an overwhelming lack of awareness about the existence of subsidies. This lack of awareness has a negative impact on the acceptance of energy price increases (Garg et al., 2016; Sharma et al., 2018; Pradiptyo et al., 2015). The lack of awareness of subsidies has also been found in Nigeria (Nwachukwu and Chike, 2011). The complexity of subsidies and subsidy reform also impacts potential support (Finch and Nelson, 2016). The hypothesis underlying the link between knowledge and support for reform is that, by learning about the negative impacts and regressive nature of structural policies, moral concerns about the impact of reforms disappear in favour of support (Lippmann, 1922; McCombs and Shaw, 1972; Gerbner, 1998). Consequently, international institutions and other practitioners have considered communication as a crucial part of energy subsidy reforms (Beaton et al., 2013; IMF, 2013; Inchauste and Victor, 2017). The connection between understanding about subsidies and support for reform has been confirmed empirically confirmed in the case of India power tariff reforms where Aklin et al. (2014) found that when consumers were made aware of the negative impacts of energy subsidies, they had a more positive attitude towards reform.

Corruption is often considered a major impediment to economic and social development (Dreher et al., 2007) and is a particularly important problem in Nigeria where it prevents the efficient provision of public services, leading to higher levels of socio-economic inequality (Ogbonna and Kalu, 2012). Corruption is a particularly important problem in the oil sector in Nigeria, including the fuel supply chain (Okonjo-Iweala and Osafo-Kwaako, 2007; Bashir, 2013; Akov, 2015). Perceptions of corruption may therefore have an important influence over support for subsidy reform since people may be willing to accept subsidy reforms if they believe that the funds saved would be used for the public good, but opposed to reform if they believe that the funds will be subject to corruption.

In addition to the above six factors, support for reform is likely to be influenced by a wide range of personal and social characteristics. Income (Acharya and Sadath, 2017), education (Garg et al., 2016; Pradiptyo et al., 2015), and geographical location (Garg et al., 2016; Pradiptyo et al., 2015) have all been shown to be relevant in explaining willingness to accept fuel subsidy reforms.

Table 1 below shows the hypotheses we wish to test for each explanatory category.

**Table 1:** Hypotheses under each explanatory category.

Explanatory Category	Hypotheses tested
Economic	1. People who pay more for fuel are more likely to support reform.
	2. People who suffer more from problems of availability are more likely to support reform
Trust in government	3. People with a higher trust in government are more likely to support reform
	4. People who support the current president (who has opposed reform) are less likely to support reform
Corruption and incapacity	5. People who believe that subsidy savings will be subject to corruption are less likely to support reform
	6. People who believe that the government does not have the capacity to spend subsidy savings well are less likely to support reform
Reciprocity & fiscal exchange	7. People who are satisfied with services are more likely to support reform
	8. People who have noticed an improvement in services are more likely to support reform
Personal norms	9. People who are more religious are more likely to support reform
Knowledge	10. People who understand how the subsidy works are more likely to support reform

## 3 Data and methodology

To test our hypotheses, we draw on a new dataset of the attitudes and perceptions of Nigerians towards tax compliance and subsidy reform. This consisted of two major quantitative surveys – a household survey and a survey of small firms conducted during July 2018, as well as a set of focus group discussions (FGD) on subsidy reform.^8^ We outline below the composition of each source of data.

### 3.1 Household/individual survey

In July 2018, a survey was conducted of 16,000 Nigerian adults across the country. A clustered, stratified, and multi-stage random selection procedure was used to achieve a nationally representative sample. The sample is also representative of Nigeria's six geopolitical zones,^9^ as well as representative of both urban and rural areas. Households were sampled from all states (with probability proportionate to their populations). Because of the small sample size in each state, results are not statistically representative at the state level.^10^ The data was collected by in-home, face-to-face personal interviews in the language with which the respondent was most comfortable. Respondents were adult Nigerian males and females aged 18 years and above. An equal number of male and female respondents were selected.^11^ They were also expected to have lived in the household for a period of not less than six months. The survey excluded non-Nigerian citizens, people aged less than 18 years, and people living in institutionalized settings. The sample was stratified to provide greater representation in urban areas: 70 per cent of individuals were selected from urban areas and 30 per cent from rural areas. Sampling weights were created to account for the design enabling us to use the entire sample of 16,000 households as a single dataset.^12^

### 3.2 Focus group discussions

Quantitative surveys can provide excellent representative data on the perceptions of individuals and firms on subsidy reform, but it is sometimes difficult to know why respondents hold the views shown. We, therefore, complemented the quantitative surveys with a set of focus group discussions (FGDs). Two FGDs on subsidy reform were held with households in each region – one with men and another with women. The FGDs were spread across both urban and rural areas.^13^ The FGDs explored: availability and delays in access to fuel; knowledge of subsidies; the incidence and relative size of the subsidies; the impact of the 2016 price increase; and attitudes towards government policy.

### 3.3 Variables

Our key dependent variable is support for reducing subsidies – a binary variable. Specifically, the question asks, ‘In your opinion, do you think it would be a good thing if the government reduced the fuel subsidy (which would increase the fuel price)?’

Our key explanatory variables follow the explanatory categories described above, as follows.^14^

1. **Economic factors.** There is a strong theoretical presumption that economic (or pocketbook) factors should influence support for reform. For example, respondents who have to pay more than the official price might be expected to feel frustrated that they are not benefiting from subsidies and be more supportive of reform in general. Similarly, respondents who have recently experienced queues or no availability of fuel might also be expected to support reform of the subsidy system.
2. **Trust in government.** Our variable about trust in government focuses on trust in the federal government, which is responsible for subsidy policy. We also include a variable on the respondent's approval of President Buhari. The president has expressed consistent opposition to petrol subsidy reform, and so we would expect supporters of the president to be less likely to support reform. However, this variable could also be endogenous, as respondents might support the president because of his stance on subsidy reform. We, therefore, estimate our model both with and without this variable.^15^
3. **Corruption and incapacity.** Respondents were asked whether, if a subsidy reform occurred, they felt that the money would be used well. Those that doubted the money would be used well were asked whether this was because they were worried about corruption or because they felt that the government lacked the capacity to implement projects well. We include these variables to explore the association between perceptions of corruption and incapacity and support for reform.
4. **Reciprocity and fiscal exchange.** The variables that we use to capture the concept of reciprocity refer to the quality of service delivery, specifically electricity and bus services. In addition, we include service improvements over the last three years. It seems plausible that the delivery of good services and improvements over time might make respondents supportive of reform measures more generally, including subsidy reform.
5. **Personal norms.** Our measure of personal social norms is whether the respondent is an active member of a religious group. The tax literature suggests that religiosity can be associated with tax compliance, but we have no clear prior belief regarding how it should affect support for subsidy reform.
6. **Knowledge and complexity.** Our measure of knowledge about subsidies is based on a question that assesses whether the respondent understands what a subsidy is and that fuel is, in fact, subsidized.

Finally, we include in our analysis a set of controls for factors that are likely to influence support for subsidy reform. These include age, level of education, employment status, gender, personal income after tax, whether they are in an urban or rural area, language group (a proxy for ethnicity), geopolitical region (including whether the respondent lives in an oil-producing state), and religion. We also include a control for whether the previous fuel price rise in 2016 had a big impact on the household or not. Appendix Table A3 provides the descriptive statistics for all the variables.

### 3.4 Model

Given that our primary outcome variable of interest is a binary indicator, we mainly estimate logistic regression models. Our primary regression equation is specified as follow:

(1) $$\text{Logit}\left(P\left(\text{SSR}=1\right)\right)=\alpha +\beta 1{\text{Economic}}_{i}+\beta 2{\text{Trust}}_{i}+\beta 3{\text{Corrupt}}_{i}+\beta 4{\text{Recip}}_{i}+\beta 5{\text{Norm}}_{i}+\beta 6{\text{Know}}_{i}+\gamma Z_{i},$$

where *i* indexes households. The dependent variable is a binary indicator of whether a household *i* thinks reducing fuel subsidy is a good thing (${\text{SSR}}_{i}$). Economic, Trust, Corrupt, Recip, Norm, and Know denotes vectors of variables in each explanatory category—economic factors, trust in government, corruption and incapacity, reciprocity and fiscal exchange, personal norms, and knowledge and understanding, respectively. In addition, we include a battery of control variables, denoted with a vector, $Z_{i}$. As robustness checks, we also estimate probit regression models and linear probability models.

One of the challenges in estimating the relationship between our explanatory factors and support for subsidy reform is that we cannot guarantee the exogeneity of the variables in the model. Problems might arise for two reasons. First, explanatory variables might be caused by support for subsidy reform, rather than the reverse (simultaneity bias); second, support for subsidy reform and explanatory variables might both be influenced by some common third variable (omitted variable bias). Unfortunately, the former is difficult to tackle since we do not have instruments for our explanatory variables, and the nature of most of our variables is such that randomization would be impossible. However, we have provided theoretical arguments about why these variables may influence support for reform and cannot see any obvious way how subsidy reform might influence the explanatory variables.

Potentially more serious is omitted variable bias. Fortunately, we have a very comprehensive dataset, and so we have addressed this by including an extensive set of controls. Nonetheless, it is possible that there may be variables which we have not included that drive both our explanatory variables and support for reform. It is therefore important to recognize that our results can only provide evidence of association and not causal relationships.

## 4 Results and discussion

### 4.1 Control variables

Table 2 shows a regression of support for subsidy reform on all the personal and regional control variables.^16^

**Table 2:** Personal and regional controls and support for subsidy reform: logistic regression estimation results.

	(1)
	est1
Urbanization	1.295^∗∗∗^
	(0.126)
Female	1.133
	(0.092)
Employed	0.862
	(0.082)
Age (25–31)	1.171^∗^
	(0.105)
Age (32–38)	1.283^∗∗^
	(0.142)
Age (39–44)	1.581^∗∗∗^
	(0.201)
Age (45–51)	1.251^∗^
	(0.165)
Age (52 above)	1.168
	(0.195)
Language group (Yoruba)	1.504^∗∗∗^
	(0.235)
Language group (Igbo)	1.236
	(0.266)
Language group (Hausa)	0.578^∗∗∗^
	(0.097)
Language group (Other)	0.768^∗^
	(0.121)
Education (Primary school)	0.993
	(0.151)
Education (Secondary school)	1.101
	(0.167)
Education (OND)	1.018
	(0.165)
Education (HND)	1.158
	(0.201)
Income (Less than 20,000 NGN)	1.060
	(0.137)
Income (20,001–40,000NGN)	1.128
	(0.160)
Income (Over 40,000NGN)	1.210
	(0.192)
Christian	0.642
	(0.270)
Muslim	0.653
	(0.279)
Traditional	0.888
	(0.585)
North east	3.422^∗∗∗^
	(1.066)
South south	0.681
	(0.185)
North central	2.172^∗∗∗^
	(0.598)
South west	0.959
	(0.275)
North west	4.552^∗∗∗^
	(1.310)
Resident in oil producing state	1.500^∗^
	(0.345)
Observations	13,532

Robust standard errors clustered at the enumeration area in parentheses.

South east region is omitted (baseline category).

We report the estimated coefficients and standard errors transformed to odds ratios.

*p < 0.10, **p < 0.05, ***p < 0.01.

The results reveal some interesting associations between the control and regional variables and support for subsidy reform. Respondents in urban areas are more likely to support reform than those in rural areas. Focus group discussions confirmed this result, with rural male respondents emphasizing the positive impact of the fuel subsidy on economic opportunity and equity more than their urban counterparts. This is in line with other studies that suggest that rural households are more opposed to price increases because of affordability, with incomes relatively lower in rural areas (Blankenship, Wong, and Urpelainen, 2019; Garg et al., 2016). This result is interesting, however, because urban households capture more of the absolute value of fuel subsidies than rural households (Kyle, 2018) and so one might expect them to be more strongly opposed to reform.

We also find that support for reform increases with age, up to around the age of 39–44, and declines thereafter, with the least support among young adults (18–24). We find no statistically significant association between gender or employment on support for reform.

The results for language and region are also interesting. Language was included as a proxy for ethnicity. However, language is strongly correlated with region, with Yoruba speakers dominating in the South West, Igbo in the South East, and Hausa in the North. Including dummies for both language and region allows one to distinguish the impact of ethnicity from that of the region. For language, the excluded category is English; for region, it is the South-East. Hence the model suggests that, relative to English speakers, Yoruba speakers are more in favour of reform, while the speakers of Hausa (and other less common languages) are more opposed. This is consistent with the traditionally commercial orientation of the Yoruba, while Hausa speakers, who dominate the Northern regions, traditionally have stronger support for state-driven development. At the same time, relative to the South-East region, all of the Northern regions (West, Central, and East) are much more in favour of reform. This may reflect the much greater problems with scarcity and higher prices in the North relative to other regions of the country.

Interestingly, respondents living in ‘derivation states’ – oil-producing states that receive an automatic allocation of oil revenue from the government – are more in favour of reform. It is not clear why this is the case. In theory, people in these states might understand that their states lose significant revenue due to the deduction of subsidy costs from revenue submitted by the national oil company to the federal government. However, this seems unlikely since people in derivation states actually have a poorer understanding of subsidies than those in non-derivation states.^17^ Moreover, focus group discussions in two derivation states provided no evidence for such an understanding.

Results for education and income are as expected, with support for reform rising with both. While this goes against their interests - since the benefits of subsidies are primarily captured by the better off – it may reflect the fact that people with higher incomes are better able to cope with price rises. Many focus group discussants shared stories about the impact of the fuel price increase in 2016. Many people had to adjust lifestyles as a result of higher prices, including radical changes such as rationing food and cutting down on health and basic transport expenditure. Poorer Nigerians are more likely to oppose reform than the better off primarily because of their lower ability to cope with the economic shock of fuel price rises.

Finally, there is no statistically significant difference in support for reform between Christians and Muslims.

### 4.2 Correlates of support for subsidy reform

Above we laid out a set of hypotheses about the factors that might influence support for subsidy reform. Table 3 shows the results of our main model which explores the association between each of our explanatory factors and support for reform.

**Table 3:** Factors that are associated with support for subsidy reform: logistic regression estimation results.

	(1)	(2)	(3)
	est1	est2	est3
Queue	1.006	1.000	1.014
	(0.146)	(0.143)	(0.145)
Paid more than the fixed price	1.679^∗∗∗^	1.698^∗∗∗^	1.658^∗∗∗^
	(0.207)	(0.215)	(0.213)
No availability	1.655^∗∗∗^	1.669^∗∗∗^	1.672^∗∗∗^
	(0.252)	(0.254)	(0.252)
General trust in federal government	1.003	1.068	0.969
	(0.106)	(0.116)	(0.103)
Approval of performance by President Buhari		0.927^∗∗^	0.918^∗∗^
		(0.032)	(0.031)
Corruption			0.568^∗∗∗^
			(0.057)
Lack of capacity			0.731^∗∗∗^
			(0.081)
Opinion about services in the area: Electricity supply	1.082^∗∗^	1.083^∗∗^	1.082^∗∗^
	(0.034)	(0.034)	(0.034)
Opinion about services in the area: Bus services	1.087^∗∗∗^	1.088^∗∗∗^	1.072^∗∗^
	(0.031)	(0.031)	(0.030)
Quality of government services provided by State Govnmt compared to 3 yrs ago	1.036	1.053	1.031
	(0.059)	(0.060)	(0.059)
Membership of religious group	1.192^∗∗^	1.185^∗∗^	1.176^∗^
	(0.103)	(0.102)	(0.101)
Understanding of subsidy granted	0.946	0.958	0.953
	(0.084)	(0.085)	(0.085)
Size of impact of 2016 fuel price increase	0.951	0.950	0.969
	(0.034)	(0.033)	(0.034)
Observations	12,213	12,185	12,185

Robust standard errors clustered at the enumeration area in parentheses.

Region dummies are included in models.

We report the estimated coefficients and standard errors transformed to odds ratios.

Results for control variables in models are omitted.

*p ¡ 0.10, **p ¡ 0.05, ***p ¡ 0.01.

Our model provides support for some of our hypotheses. We suggested that economic variables were likely to be an important factor in determining support for reform. This appears to be confirmed. Respondents that had to pay more than the official price are much more likely to support reform^18^; the same is true of respondents that have experienced fuel not being available at all within the month prior to the survey. Focus group discussions confirmed large-scale discontent with supply disruptions, especially around religious holidays.

We also hypothesized that trust in the Federal government would be key to achieving reform. There is surprisingly little support for this, although this may reflect the fact that the vast majority (75%) of respondents have little or no trust in government at all.^19^ We also control for respondent's approval of President Buhari. Here we obtain a strong result – respondents who approve of the president's performance are strongly opposed to subsidy reform, reflecting the publicly-stated position of the president. However, as noted above, it is possible that their support for the president is because of his position on subsidy reform, making the variable endogenous. Our first model in Table 3 therefore omits this variable.

As expected, a belief that the government is corrupt or incapable of implementing programs effectively is strongly associated with opposition to subsidy reform. This is consistent with the findings from the focus groups where allegations of corruption and incompetence by the government were common.

Variables representing fiscal reciprocity appear to be strongly associated with support for reform. In areas where services (electricity and bus services) are better, support for reform is stronger, although recent improvements in service delivery do not appear to have a strong influence on support for reform. Since electricity provision in Nigeria is notoriously weak, many households receive electricity from generators. Focus group discussants confirmed that these generators run on petrol. A better supply of grid-based electricity would reduce the need to consume petrol in a generator. This may explain why improvements in electricity increase the acceptance of subsidy reforms that would raise the price of fuel. Alternatively, it may simply reflect greater trust in reform by those that experience more competent government service delivery.

Personal norms also seem to influence support for reform. In parallel to the tax literature, respondents who are active participants in religious groups are also more supportive of reform. This might be because the personal beliefs that are associated with religiosity are correlated with those that support reform, or it may be that being part of a religious group provides a degree of social protection, making respondents more willing to entertain reforms.

To our surprise, knowing of the existence of the subsidy and how it works does not appear to have any influence on whether one supports subsidy reform. Subsidy reform, it would appear, is something that people have made up their minds about regardless of whether they know what a subsidy is.

Finally, it is possible that attitudes towards subsidy reform are influenced by the experience of previous subsidy reforms.^20^ We, therefore, included a variable about whether the previous fuel price rise in 2016 had a big impact on the household or not. As expected, we find a negative correlation with support for reform, but the effect is not statistically significant.

To check that our results are not due to our particular methodology, we also estimated all our models using a Probit model and with OLS. The results for the comprehensive model are presented in Appendix Tables A13 and A14. The results are qualitatively the same.

## 5 Conclusions and policy implications

Given the scale of energy subsidies and the damage that such subsidies cause, reforming them is an important policy objective, particularly in countries like Nigeria, where they represent a significant share of public resources. However, achieving such reforms is difficult, not least because subsidies have widespread public support. Understanding what drives support for reform is therefore useful since it potentially enables policymakers to design more effective reform strategies.

We have outlined a set of factors that theory and the existing literature would suggest are likely to be associated with support for reform. Exploiting a large new dataset in Nigeria, we have tested these hypotheses and find broad confirmation of several of our hypotheses. In particular, traditional economic factors, such as price and availability, appear to be associated with support for reform; where customers are charged more than the regulated price, or where they have experienced a lack of fuel, they tend to be more in favour of reform. On the other hand, if Nigerians believe that savings from reducing fuel subsidies will not be used appropriately because of corruption or because the government is not capable of running programs efficiently, then their support for subsidy reforms appears to dwindle. Indeed, corruption reduces support for reform more than any other factor. While general trust in government does not appear to be associated with support for reform, the delivery of reasonable national and local services is, supporting the idea that building the ‘social contract’ is key to reform. Personal norms, such as active participation in religious groups, also appear correlated with support for reform. Intriguingly, actual knowledge about subsidies is not – people appear to form their opinions on the issue regardless of their understanding of it.

As noted above, there is surprisingly little rigorous research on the drivers of public opinion about such reforms in developing countries. Future research could usefully develop in three ways. First, to our knowledge, this is the first rigorous assessment of the determinants of support for subsidy reform in Africa; it would be useful to replicate this work in other countries facing significant subsidies (McCulloch and Dom (2019) show that this includes around a quarter of the countries in sub-Saharan Africa and dozens of countries in other regions). Second, it would be interesting to delve into the reasons behind some of our results – for example, why and how do religious norms influence support for subsidy reform? Third, given our surprising result on knowledge, it would be useful to replicate this study with a strong, randomized communications intervention. In this way, concrete evidence could be obtained about what sort of messages are most likely to be effective in shifting peoples’ opinions on issues of subsidy reform and how these vary by context.

Our results also provide some pointers for policymakers designing reforms. Policy should strengthen the voice of those who have a reason to support reform, such as those experiencing high prices or shortages. At the same time, wider measures to build trust in government and to strengthen the social contract, such as actions to tackle corruption and improve the delivery of services, are also likely to help in making subsidy reforms more feasible. In summary, perhaps it is not surprising that people are more likely to support reform of fuel subsidies when the government shows that it can be trusted to deliver fuel and other services in a clean and competent way.

## CRediT authorship contribution statement

**Neil McCulloch:** Conceptualization, Data curation, Formal analysis, Funding acquisition, Investigation, Methodology, Project administration, Resources, Software, Supervision, Validation, Visualization, Roles, Writing – original draft, Writing – review & editing. **Tom Moerenhout:** Conceptualization, Data curation, Formal analysis, Funding acquisition, Investigation, Methodology, Project administration, Resources, Software, Supervision, Validation, Visualization, Roles, Writing – original draft, Writing – review & editing. **Joonseok Yang:** Conceptualization, Data curation, Formal analysis, Funding acquisition, Investigation, Methodology, Project administration, Resources, Software, Supervision, Validation, Visualization, Roles, Writing – original draft, Writing – review & editing.

## Declaration of competing interest

The authors declare that they have no known competing financial interests or personal relationships that could have appeared to influence the work reported in this paper.

## Footnotes

1 The IEA uses a price-gap approach to calculate energy subsidies, which include both fossil fuel subsidies and subsidies to electricity, much of which is generated from fossil fuels.

2 Defined as living below $1.90 in 2011 PPP dollars.

3 McCulloch (2017) estimates that globally bilateral donors provided only $35 million in direct efforts to remove energy subsidies – a tiny fraction of all aid.

4 Estimated by the World Bank to be $2.2 billion – see Hafez (2018).

5 Previously the government paid the difference to private marketers out of the government budget. Currently the government mandates the national oil company, NNPC, to undertake all imports, and they deduct the ‘under-recovery’ from the oil revenue that they remit to the government. Subsidies used to be provided to diesel and kerosene as well, but are now only provided to petrol.

6 The government has attempted to ‘reform’ subsidies numerous times. However, this has almost always been done simply by increasing to a new fixed price. As a result of inflation, currency depreciation and changes in international oil prices, subsidies always re-emerge.

7 This said, we acknowledge that fiscal reciprocity around taxation does differ from that around subsidies. In particular, people often regard services as a right, and view taxation as a reasonable obligation if those services are delivered. In contrast, many people regard subsidies as a right, and so do not always see the provision of services or other public goods as a reasonable form of compensation for their removal.

8 The data collection process was coordinated by Neil McCulloch and Tom Moerenhout and funded by the Bill and Melinda Gates Foundation in support of the work of the Nigeria Economic Summit Group. The Nigeria-based survey firm Practical Sampling International conducted the data collection. The survey and focus group discussions were piloted in May 2018, and the data collection exercise was conducted in July and August 2018. The firm survey did not contain questions on subsidy reform. The full data and documentation are available on https://www.ictd.ac/dataset/␣nesg-nigeria-tax-subsidy-perception-dataset/.

9 North-Central, North-East, North-West, South-East, South-South and South-West.

10 In addition, six states were over-sampled to provide state level representativeness (Abia, Bauchi, Kano, Nassarawa, Ogun, Rivers) – see McCulloch and Moerehhout (2019) for details.

11 After determining whether a man or women would be selected in each household, the selection of respondent was done randomly from all the adult household members of that gender.

12 See the Field, Technical and Methodology Report available from https://www.ictd.ac/dataset/nesg-nigeria-tax-subsidy-perception-dataset/ for details.

13 Separate FGDs were also held to explore tax perceptions – see (McCulloch et al., 2019) for details.

14 Appendix A1 shows the actual questions asked for each of the variables used in the analysis. Full documentation for the survey, including the survey instruments is available at https://www.ictd.ac/dataset/nesg-nigeria-tax-subsidy-perception-dataset/.

16 Results are presented as odds ratios, so a value of greater than one indicates an increase in the odd-ratio of support for subsidy reform, while a figure of less than one shows a decrease.

17 In non-derivation states, 33% of respondents understood that the price at which the government purchases petrol from the oil companies is higher than the price of petrol sold to the public; in derivation states, only 23% of respondents understood this.

18 This variable measures whether the respondent says that they paid more than the official price. As a robustness check, we also calculated the gap between the price they actually paid and the official price to see if this was also associated with support for reform; we find rather weak evidence for this, suggesting that support for reform is higher when people perceive that they are paying more than the official price, but does not necessarily depend on the price they actually pay (the results are presented in Appendix Table A10).

19 Our robustness tests also looked at trust in state and local government, with the same result – see Appendix Table A11.

20 We are grateful to one reviewer for suggesting this to us.

## Footnotes

15 One reviewer pointed out that this could also be true of trust i.e. the government's position on subsidy reform might influence whether people trust the government. Alternatively, respondents may hold an ideological view that influences both trust in government and support for subsidy reform. We have attempted to account for this in our robustness tests by using trust in state and local government, which are likely to be correlated with trust in Federal government, but not with support for subsidy reform.
