# Supplementary material for: Fuel subsidy reform and the social contract in Nigeria: A micro-economic analysis
Source: Energy Policy. 2021 Sep;156:None. doi: 10.1016/j.enpol.2021.112336 (PMC8359919; doi:10.1016/j.enpol.2021.112336)
Supplement: Multimedia component 2 [file mmc2.pdf]

Fuel subsidy reform and the social contract in Nigeria: a micro-economic analysis

Supporting Information

Contents

A1 Actual questions asked for each of the variables used in the analysis APP-2

A2 Descriptive statistics APP-3

A3 Main estimation results by explanatory category APP-5

A4 Robustness checks: Impact of patial price variation on support for reform APP-7

A5 Robustness checks: Estimations using a variable about trust in local / state governments instead of federal government APP-8

A6 Robustness checks: Estimations using a variable about opinion toward 'tax for development' APP-9

A7 Robustness checks: Probit estimation results APP-10

A8 Robustness checks: OLS estimation results APP-11

A9 Robustness checks: Estimation results using a binary indicator for big impact of 2016 fuel price increase APP-12

A1 Actual questions asked for each of the variables used in the analysis

Variable Name	Question	Response	Specification of variable
Queue	Did you or others in your household encounter any of the following difficulties when buying PMS/Petrol/Fuel in the past month? Having to queue for a long time	Yes (1), No (2), Don't' know / Refused (98/99)	Coded 1 for "Yes" and 0 otherwise (omitting "don't know/refused")
Paid more than the fixed price	Did you or others in your household encounter any of the following difficulties when buying PMS/Petrol/Fuel in the past month? Having to pay more than the official price	Yes (1), No (2), Don't' know / Refused (98/99)	Coded 1 for "Yes" and 0 otherwise (omitting "don't know/refused")
No availability	Did you or others in your household encounter any of the following difficulties when buying PMS/Petrol/Fuel in the past month? PMS/Petrol/Fuel not available at all	Yes (1), No (2), Don't' know / Refused (98/99)	Coded 1 for "Yes" and 0 otherwise (omitting "don't know/refused")
General trust in federal government	In your opinion, do you think the Federal Government acts more for the good of the Nigerian people or more for its own interests?	Acts more for the good of the Nigerian people (1), Acts more for its own interests? (2), Don't' know / Refused (98/99)	Coded 1 for "for the good of the Nigerian people" and 0 otherwise (omitting "don't know/refused")
Approval of performance by President Buhari	Generally speaking, to what extent do you approve or disapprove of the performance of the following people? President Buhari	Strongly disapprove (1) --- Strongly Approve (5), , Don't' know / Refused (98/99)	Coded as an ordinal variable (from 1 to 5 where the higher value denotes the higher level of trust, omitting "don't know/refused")
Corruption	How confident are you that the money saved by the government from the reduction of fuel subsidy will be used appropriately for the greater good of public interest? (for the respondents who said "either doubtful or very doubtful," we asked "What is the main reason?"	Because the money will be corrupted (1), Because the government is not capable of running programs efficiently (2), Don't' know / Refused (98/99)	Coded 1 for "Because the money will be corrupted" and 0 for the responses that (very) confident that the money will be used appropriately, omitting "don't know/refused")
Lack of capacity	How confident are you that the money saved by the government from the reduction of fuel subsidy will be used appropriately for the greater good of public interest? (for the respondents who said "either doubtful or very doubtful," we asked "What is the main reason?"	Because the money will be corrupted (1), Because the government is not capable of running programs efficiently (2), Don't' know / Refused (98/99)	Coded 1 for "Because the government is not capable of running programs efficiently" and 0 for the responses that (very) confident that the money will be used appropriately, omitting "don't know/refused")
Opinion about services in the area: Electricity supply	Some people are satisfied with the quality of public services in this area. Others are dissatisfied with the public services. What is your opinion about the following services in this area? Electricity supply	Very dissatified (1) --- Very satisfied (5), no services in my area (0), Don't' know / Refused (98/99)	Coded as an ordinal variable (from 1 to 5 where the higher value denotes the higher level of satisfaction, omitting "don't know/refused")
Opinion about services in the area: Bus services	Some people are satisfied with the quality of public services in this area. Others are dissatisfied with the public services. What is your opinion about the following services in this area? Bus services	Very dissatified (1) --- Very satisfied (5), no services in my area (0), Don't' know / Refused (98/99)	Coded as an ordinal variable (from 1 to 5 where the higher value denotes the higher level of satisfaction, omitting "don't know/refused")
Quality of government services provided by State Govnmt compared to 3 yrs ago	What do you in general think of the quality of government services provided by this state Government today compared to three years ago?	Worse than before (1), About the same (2), Better than before (3), Don't' know / Refused (98/99)	Coded as an ordinal variable (from 1 to 3 where the higher value denotes the higher level of satisfaction, omitting "don't know/refused")
Membership of religious group	Do you belong/affiliated to any religious group/association?	Yes (1), No (2)	Coded 1 for "Yes" and 0 otherwise
Understanding of subsidy granted	Please explain that the official price of PMS/Petrol/Fuel is N 145 per litre. Explain also that in order to meet domestic demand, the government of Nigeria buys PMS/Petrol and other fuels from oil companies at home and abroad. After that, the government distributes and sells them through retail Petrol/Filling stations. The government also determines the value of the fuel price sold to the public through retail Petrol/Filing stations.	Price at which the government purchases PMS/Petrol/Fuel from the oil companies is (lower (1) / same (2) / higher (3)) than the price of PMS/Petrol/Fuel sold to the public at N 145	Coded 1 for "Higher (3)" and 0 otherwise
Deviation from N145	During the last one week, what was the total amount spent by your whole household on these types of fuel? PMS (Petrol/Fuel) - How much Naira spent [Put 0 if you did not purchase this fuel during the last week]	Naira spent	We substracted 145 from the amount of Naira spent for PMS
General trust in state government	Do you think the state Government in this area acts more for the good of the local people or more for its own interests?	Acts more for the good of the people in this State (1), Acts more for its own interests? (2), Don't' know / Refused (98/99)	Coded 1 for "for the good of the people in this state" and 0 otherwise (omitting "don't know/refused")
General trust in local government	Do you think the local Government in this area acts more for the good of the local people or more for its own interests?	Acts more for the good of the local people (1), Acts more for its own interests? (2), Don't' know / Refused (98/99)	Coded 1 for "for the good of the local people" and 0 otherwise (omitting "don't know/refused")
Tax for development	I am now going to read you two statements about taxation. After I have read both statements please tell me how much you agree or disagree with each statement. a) Citizens must pay their taxes to the Government in order for the country to develop	Agree (3), Neither Agree nor Disagree (2), Disagree (1), Don't' know / Refused (98/99)	Coded as an ordinal variable (from 1 to 3, omitting "don't know/refused")
Size of impact of 2016 fuel price increase	How big was the impact of the 2016 fuel price increase on you and your household?	No impact at all (1), Little impact (2), Medium impact (3), Big impact (4), Very big impact (5)	Coded as an ordinal variable (from 1 to 5 where the higher value denotes the larger impact)

Table A1: Actual questions asked for each of the variables used in the analysis and coding schemes that we applied to create the variables.

A2 Descriptive statistics

Variable	Mean	Std. Dev.	Min.	Max.	N
Good to reduce fuel subsidy	0.314	0.464	0	1	16228
Narrative group (Control group)	0.2	0.4	0	1	16228
Narrative group (Income distribution)	0.2	0.4	0	1	16228
Narrative group (Alternative expenditure)	0.2	0.4	0	1	16228
Narrative group (Link subsidy and availability)	0.2	0.4	0	1	16228
Narrative group (Subsidy and oil nationalism)	0.2	0.4	0	1	16228
Queue	0.088	0.283	0	1	16228
Paid more than the fixed price	0.109	0.312	0	1	16228
No availability	0.077	0.266	0	1	16228
General trust in federal government	0.306	0.461	0	1	15738
Approval of performance by President Buhari	1.437	1.494	0	4	16156
Opinion about services in the area: Electricity supply	2.308	1.55	0	5	16170
Opinion about services in the area: Bus services	1.937	1.623	0	5	16046
Quality of government services provided by State Govnmt compared to 3 yrs ago	1.73	0.779	1	3	14899
Tax for development	2.408	0.830	1	3	15947
Membership of religious group	0.414	0.493	0	1	16228
Understanding of subsidy granted	0.322	0.467	0	1	16228
Urbanization	0.700	0.458	0	1	16228
Female	0.5	0.5	0	1	16228
Employed	1.494	0.5	1	2	16096
Age (18-24)	0.223	0.416	0	1	16228
Age (25-31)	0.347	0.476	0	1	16228
Age (32-38)	0.207	0.405	0	1	16228
Age (39-44)	0.101	0.301	0	1	16228
Age (45-51)	0.067	0.251	0	1	16228
Age (52 above)	0.055	0.227	0	1	16228
Language group (English)	0.241	0.428	0	1	16221
Language group (Yoruba)	0.152	0.359	0	1	16221
Language group (Igbo)	0.117	0.322	0	1	16221
Language group (Hausa)	0.349	0.477	0	1	16221
Language group (Other)	0.14	0.348	0	1	16221
Education (No schooling)	0.08	0.271	0	1	16189
Education (Primary school)	0.1	0.301	0	1	16189
Education (Secondary school)	0.451	0.498	0	1	16189
Education (OND)	0.184	0.388	0	1	16189
Education (HND)	0.184	0.388	0	1	16189
Income (None)	0.22	0.414	0	1	13597
Income (Less than 20,000 NGN)	0.361	0.48	0	1	13597
Income (20,001-40,000NGN)	0.247	0.431	0	1	13597
Income (Over 40,000NGN)	0.172	0.377	0	1	13597
No religion	0.002	0.045	0	1	16228
Christian	0.576	0.494	0	1	16228
Muslim	0.417	0.493	0	1	16228
Traditional	0.003	0.055	0	1	16228
South east	0.136	0.343	0	1	16228
North east	0.147	0.354	0	1	16228
South south	0.157	0.363	0	1	16228
North central	0.153	0.36	0	1	16228
South west	0.186	0.389	0	1	16228
North west	0.221	0.415	0	1	16228
Resident in oil producing state	0.251	0.434	0	1	16228

Table A2: Descriptive Statistics

Variable	Mean	Std. Dev.	Min.	Max.	N
Good to reduce fuel subsidy	0.314	0.464	0	1	16228
Narrative group (Control group)	0.2	0.4	0	1	16228
Narrative group (Income distribution)	0.2	0.4	0	1	16228
Narrative group (Alternative expenditure)	0.2	0.4	0	1	16228
Narrative group (Link subsidy and availability)	0.2	0.4	0	1	16228
Narrative group (Subsidy and oil nationalism)	0.2	0.4	0	1	16228
Queue	0.088	0.283	0	1	16228
Paid more than the fixed price	0.109	0.312	0	1	16228
No availability	0.077	0.266	0	1	16228
General trust in federal government	0.306	0.461	0	1	15738
Approval of performance by President Buhari	1.437	1.494	0	4	16156
Opinion about services in the area: Electricity supply	2.308	1.55	0	5	16170
Opinion about services in the area: Bus services	1.937	1.623	0	5	16046
Quality of government services provided by State Govnmt compared to 3 yrs ago	1.73	0.779	1	3	14899
Tax for development	2.408	0.830	1	3	15947
Membership of religious group	0.414	0.493	0	1	16228
Understanding of subsidy granted	0.322	0.467	0	1	16228
Urbanization	0.700	0.458	0	1	16228
Female	0.5	0.5	0	1	16228
Employed	1.494	0.5	1	2	16096
Age (18-24)	0.223	0.416	0	1	16228
Age (25-31)	0.347	0.476	0	1	16228
Age (32-38)	0.207	0.405	0	1	16228
Age (39-44)	0.101	0.301	0	1	16228
Age (45-51)	0.067	0.251	0	1	16228
Age (52 above)	0.055	0.227	0	1	16228
Language group (English)	0.241	0.428	0	1	16221
Language group (Yoruba)	0.152	0.359	0	1	16221
Language group (Igbo)	0.117	0.322	0	1	16221
Language group (Hausa)	0.349	0.477	0	1	16221
Language group (Other)	0.14	0.348	0	1	16221
Education (No schooling)	0.08	0.271	0	1	16189
Education (Primary school)	0.1	0.301	0	1	16189
Education (Secondary school)	0.451	0.498	0	1	16189
Education (OND)	0.184	0.388	0	1	16189
Education (HND)	0.184	0.388	0	1	16189
Income (None)	0.22	0.414	0	1	13597
Income (Less than 20,000 NGN)	0.361	0.48	0	1	13597
Income (20,001-40,000NGN)	0.247	0.431	0	1	13597
Income (Over 40,000NGN)	0.172	0.377	0	1	13597
No religion	0.002	0.045	0	1	16228
Christian	0.576	0.494	0	1	16228
Muslim	0.417	0.493	0	1	16228
Traditional	0.003	0.055	0	1	16228
South east	0.136	0.343	0	1	16228
North east	0.147	0.354	0	1	16228
South south	0.157	0.363	0	1	16228
North central	0.153	0.36	0	1	16228
South west	0.186	0.389	0	1	16228
North west	0.221	0.415	0	1	16228
Resident in oil producing state	0.251	0.434	0	1	16228

Table A3: Descriptive Statistics

A3 Main estimation results by explanatory category

	(1) est1	(2) est2	(3) est3	(4) est4
Good to reduce fuel subsidy				
Queue	1.581***			1.049
	(0.205)			(0.150)
Paid more than the fixed price		2.001***		1.666***
		(0.223)		(0.202)
No availability			2.160***	1.734***
			(0.271)	(0.257)
Observations	13532	13532	13532	13532

Robust standard errors clustered at the enumeration area in parentheses.
Region dummies are included in models.
We report the estimated coefficients and standard errors transformed to odds ratios.
Results for control variables in models are omitted.
*$p < 0.10$, ** $p < 0.05$, *** $p < 0.01$*

Table A4: Main estimation results by explanatory category - 'Economic factors'

	(1) est1	(2) est2	(3) est3
Good to reduce fuel subsidy			
General trust in federal government	1.084		1.191
	(0.113)		(0.127)
Approval of performance by President Buhari		0.947*	0.927**
		(0.031)	(0.031)
Observations	13209	13485	13178

Robust standard errors clustered at the enumeration area in parentheses.
Region dummies are included in models.
We report the estimated coefficients and standard errors transformed to odds ratios.
Results for control variables in models are omitted.
*$p < 0.10$, ** $p < 0.05$, *** $p < 0.01$*

Table A5: Main estimation results by explanatory category - 'Trust in government'

	(1) est1	(2) est2	(3) est3
Good to reduce fuel subsidy			
Corruption	0.643***		0.586***
	(0.055)		(0.060)
Lack of capacity		1.054	0.801*
		(0.106)	(0.093)
Observations	13532	13532	13532

Robust standard errors clustered at the enumeration area in parentheses.
Region dummies are included in models.
We report the estimated coefficients and standard errors transformed to odds ratios.
Results for control variables in models are omitted.
*$p < 0.10$, ** $p < 0.05$, *** $p < 0.01$*

Table A6: Main estimation results by explanatory category - 'Corruption and incapacity'

	(1) est1	(2) est2	(3) est3	(4) est4
Good to reduce fuel subsidy				
Opinion about services in the area: Electricity supply	1.124***			1.087***
	(0.034)			(0.034)
Opinion about services in the area: Bus services		1.104***		1.084***
		(0.031)		(0.032)
Quality of government services provided by State Govnmt compared to 3 yrs ago			1.022	1.021
			(0.057)	(0.057)
Observations	13494	13392	12509	12386

Robust standard errors clustered at the enumeration area in parentheses.
Region dummies are included in models.
We report the estimated coefficients and standard errors transformed to odds ratios.
Results for control variables in models are omitted.
**p < 0.10, ** p < 0.05, *** p < 0.01*

Table A7: Main estimation results by explanatory category - ‘Reciprocity and fiscal exchange’

	(1) est1
Good to reduce fuel subsidy	
Membership of religious group	1.233**
	(0.103)
Observations	13532

Robust standard errors clustered at the enumeration area in parentheses.
Region dummies are included in models.
We report the estimated coefficients and standard errors transformed to odds ratios.
Results for control variables in models are omitted.
**p < 0.10, ** p < 0.05, *** p < 0.01*

Table A8: Main estimation results by explanatory category - ‘Personal norms’

	(1) est1
Good to reduce fuel subsidy	
Understanding of subsidy granted	0.950
	(0.085)
Observations	13532

Robust standard errors clustered at the enumeration area in parentheses.
Region dummies are included in models.
We report the estimated coefficients and standard errors transformed to odds ratios.
Results for control variables in models are omitted.
**p < 0.10, ** p < 0.05, *** p < 0.01*

Table A9: Main estimation results by explanatory category - ‘Knowledge and complexity’

A4 Robustness checks: Impact of patial price variation on support for reform

	(1)<br>est1	(2)<br>est2	(3)<br>est3	(4)<br>est4	(5)<br>est5	(6)<br>est6
Good to reduce fuel subsidy						
Queue	0.839	0.842	0.857	0.847	0.850	0.864
	(0.145)	(0.142)	(0.142)	(0.146)	(0.142)	(0.142)
Deviation from N145	1.005	1.005	1.005*			
	(0.003)	(0.003)	(0.003)			
Deviation from N145 (squared)	1.000	1.000	1.000			
	(0.000)	(0.000)	(0.000)			
Deviation from N145 (logged)				1.050	1.051	1.055
				(0.047)	(0.047)	(0.047)
No availability	2.179***	2.207***	2.194***	2.171***	2.201***	2.189***
	(0.337)	(0.340)	(0.331)	(0.337)	(0.340)	(0.332)
General trust in federal government	0.959	1.071	0.970	0.957	1.070	0.969
	(0.115)	(0.130)	(0.115)	(0.115)	(0.130)	(0.115)
Approval of performance by President Buhari		0.885***	0.877***		0.884***	0.876***
		(0.037)	(0.037)		(0.037)	(0.037)
Corruption			0.534***			0.536***
			(0.067)			(0.068)
Lack of capacity			0.758**			0.752**
			(0.107)			(0.106)
Opinion about services in the area: Electricity supply	1.097**	1.100***	1.091**	1.094**	1.097**	1.088**
	(0.040)	(0.040)	(0.040)	(0.040)	(0.040)	(0.040)
Opinion about services in the area: Bus services	1.069*	1.068*	1.052	1.070*	1.069*	1.052
	(0.039)	(0.039)	(0.039)	(0.040)	(0.039)	(0.039)
Quality of government services provided by State Govnmt compared to 3 yrs ago	1.134*	1.158**	1.119	1.135*	1.159**	1.119*
	(0.077)	(0.079)	(0.077)	(0.077)	(0.079)	(0.077)
Tax for development	1.185**	1.191**	1.173**	1.176**	1.183**	1.164**
	(0.082)	(0.083)	(0.082)	(0.081)	(0.082)	(0.081)
Membership of religious group	1.295**	1.270**	1.253**	1.301**	1.275**	1.259**
	(0.140)	(0.139)	(0.138)	(0.141)	(0.140)	(0.139)
Understanding of subsidy granted	1.042	1.067	1.074	1.040	1.065	1.072
	(0.112)	(0.115)	(0.116)	(0.112)	(0.115)	(0.116)
Observations	6278	6270	6270	6278	6270	6270

Robust standard errors clustered at the enumeration area in parentheses.
Region dummies are included in models.
We report the estimated coefficients and standard errors transformed to odds ratios.
Results for control variables in models are omitted.
*$p < 0.10$, ** $p < 0.05$, *** $p < 0.01$*

Table A10: The impact of spatial price variation on support for reform: Logistic regression estimations results adding deviations from the official price instead of 'overpay' variable

A5 Robustness checks: Estimations using a variable about trust in local / state governments instead of federal government

	(1) est1	(2) est2	(3) est3	(4) est4	(5) est5	(6) est6
Good to reduce fuel subsidy						
Queue	1.042	1.037	1.052	1.032	1.023	1.047
	(0.152)	(0.149)	(0.150)	(0.153)	(0.150)	(0.152)
Paid more than the fixed price	1.673***	1.695***	1.653***	1.696***	1.720***	1.678***
	(0.209)	(0.217)	(0.214)	(0.212)	(0.220)	(0.217)
No availability	1.619***	1.631***	1.643***	1.643***	1.657***	1.666***
	(0.246)	(0.249)	(0.247)	(0.250)	(0.253)	(0.251)
General trust in state government	1.064	1.109	1.016			
	(0.111)	(0.117)	(0.106)			
General trust in local government				0.955	0.985	0.899
				(0.096)	(0.098)	(0.088)
Approval of performance by President Buhari		0.925**	0.912***		0.926**	0.912***
		(0.031)	(0.030)		(0.031)	(0.030)
Corruption			0.568***			0.560***
			(0.057)			(0.057)
Lack of capacity			0.733***			0.736***
			(0.085)			(0.086)
Opinion about services in the area: Electricity supply	1.080**	1.081**	1.080**	1.088***	1.091***	1.089***
	(0.034)	(0.034)	(0.034)	(0.034)	(0.034)	(0.034)
Opinion about services in the area: Bus services	1.084***	1.086***	1.070**	1.090***	1.092***	1.076***
	(0.031)	(0.031)	(0.031)	(0.031)	(0.031)	(0.031)
Quality of government services provided by State Govnmt compared to 3 yrs ago	1.034	1.053	1.031	1.054	1.076	1.050
	(0.058)	(0.060)	(0.058)	(0.059)	(0.061)	(0.060)
Membership of religious group	1.189**	1.182*	1.174*	1.189**	1.182*	1.176*
	(0.102)	(0.101)	(0.101)	(0.103)	(0.102)	(0.102)
Understanding of subsidy granted	0.944	0.960	0.954	0.933	0.949	0.944
	(0.085)	(0.087)	(0.087)	(0.084)	(0.086)	(0.086)
Observations	12193	12164	12164	11987	11958	11958

Robust standard errors clustered at the enumeration area in parentheses.
Region dummies are included in models.
We report the estimated coefficients and standard errors transformed to odds ratios.
Results for control variables in models are omitted.
**p < 0.10, ** p < 0.05, *** p < 0.01*

Table A11: Logistic regression estimations results using indicators of trust in local / state governments

A6 Robustness checks: Estimations using a variable about opinion toward 'tax for development'

	(1) est1	(2) est2	(3) est3
Good to reduce fuel subsidy			
Queue	1.002	0.995	1.009
	(0.146)	(0.144)	(0.144)
Paid more than the fixed price	1.688***	1.708***	1.663***
	(0.211)	(0.219)	(0.216)
No availability	1.657***	1.674***	1.681***
	(0.253)	(0.256)	(0.255)
General trust in federal government	1.007	1.074	0.966
	(0.108)	(0.118)	(0.104)
Approval of performance by President Buhari		0.924**	0.915***
		(0.031)	(0.031)
Corruption			0.562***
			(0.057)
Lack of capacity			0.710***
			(0.078)
Opinion about services in the area: Electricity supply	1.083**	1.084**	1.083**
	(0.034)	(0.034)	(0.034)
Opinion about services in the area: Bus services	1.069**	1.070**	1.054*
	(0.030)	(0.030)	(0.029)
Quality of government services provided by State Govnmt compared to 3 yrs ago	1.026	1.043	1.020
	(0.058)	(0.060)	(0.058)
Tax for development	1.115**	1.127**	1.109*
	(0.061)	(0.061)	(0.060)
Membership of religious group	1.184**	1.178*	1.173*
	(0.101)	(0.100)	(0.100)
Understanding of subsidy granted	0.941	0.952	0.952
	(0.085)	(0.086)	(0.086)
Observations	12107	12082	12082

Robust standard errors clustered at the enumeration area in parentheses.
Region dummies are included in models.
We report the estimated coefficients and standard errors transformed to odds ratios.
Results for control variables in models are omitted.
**p < 0.10, ** p < 0.05, *** p < 0.01*

Table A12: Factors that influence support for subsidy reform: Logistic regression estimation results. We added a variable about opinion toward 'tax for development.' The results for control variables are omitted in the table.

A7 Robustness checks: Probit estimation results

	(1) est1	(2) est2	(3) est3
Good to reduce fuel subsidy			
Queue	1.008	1.005	1.011
	(0.090)	(0.089)	(0.089)
Paid more than the fixed price	1.367***	1.377***	1.358***
	(0.104)	(0.107)	(0.106)
No availability	1.356***	1.362***	1.362***
	(0.125)	(0.126)	(0.125)
General trust in federal government	1.015	1.053	0.985
	(0.066)	(0.069)	(0.063)
Approval of performance by President Buhari		0.957**	0.952**
		(0.019)	(0.019)
Corruption			0.705***
			(0.042)
Lack of capacity			0.816***
			(0.056)
Opinion about services in the area: Electricity supply	1.049**	1.050**	1.049**
	(0.020)	(0.020)	(0.020)
Opinion about services in the area: Bus services	1.051***	1.051***	1.042**
	(0.018)	(0.018)	(0.018)
Quality of government services provided by State Govnmt compared to 3 yrs ago	1.028	1.036	1.021
	(0.035)	(0.035)	(0.035)
Membership of religious group	1.110**	1.106**	1.101*
	(0.057)	(0.056)	(0.056)
Understanding of subsidy granted	0.970	0.976	0.976
	(0.051)	(0.052)	(0.052)
Observations	12213	12185	12185

Robust standard errors clustered at the enumeration area in parentheses.
Region dummies are included in models.
We report the estimated coefficients and standard errors transformed to odds ratios.
Results for control variables in models are omitted.
**p < 0.10, ** p < 0.05, *** p < 0.01*

Table A13: Factors that influence support for subsidy reform: Probit regression estimation results. The results for control variables are omitted in the table.

A8 Robustness checks: OLS estimation results

	(1) est1	(2) est2	(3) est3
Queue	1.007	1.006	1.008
	(0.032)	(0.032)	(0.031)
Paid more than the fixed price	1.117***	1.119***	1.113***
	(0.031)	(0.031)	(0.031)
No availability	1.117***	1.118***	1.118***
	(0.039)	(0.038)	(0.038)
General trust in federal government	1.004	1.018	0.994
	(0.022)	(0.023)	(0.022)
Approval of performance by President Buhari		0.985**	0.983**
		(0.007)	(0.007)
Corruption			0.888***
			(0.018)
Lack of capacity			0.929***
			(0.022)
Opinion about services in the area: Electricity supply	1.017**	1.017**	1.017**
	(0.007)	(0.007)	(0.007)
Opinion about services in the area: Bus services	1.017***	1.017***	1.014**
	(0.006)	(0.006)	(0.006)
Quality of government services provided by State Govnmt compared to 3 yrs ago	1.009	1.012	1.007
	(0.011)	(0.011)	(0.011)
Membership of religious group	1.034**	1.033*	1.032*
	(0.018)	(0.018)	(0.017)
Understanding of subsidy granted	0.987	0.989	0.988
	(0.018)	(0.018)	(0.018)
Observations	12213	12185	12185

Robust standard errors clustered at the enumeration area in parentheses.
Region dummies are included in models.
We report the estimated coefficients and standard errors transformed to odds ratios.
Results for control variables in models are omitted.
**p < 0.10, ** p < 0.05, *** p < 0.01*

Table A14: Factors that influence support for subsidy reform: OLS regression estimation results. The results for control variables are omitted in the table.

A9 Robustness checks: Estimation results using a binary indicator for big impact of 2016 fuel price increase

	(1)<br>est1	(2)<br>est2	(3)<br>est3
Good to reduce fuel subsidy			
Queue	1.008	1.003	1.017
	(0.146)	(0.144)	(0.145)
Paid more than the fixed price	1.679***	1.697***	1.658***
	(0.208)	(0.216)	(0.214)
No availability	1.650***	1.664***	1.667***
	(0.250)	(0.253)	(0.250)
General trust in federal government	1.009	1.074	0.972
	(0.108)	(0.118)	(0.104)
Approval of performance by President Buhari		0.927**	0.918**
		(0.032)	(0.031)
Corruption			0.564***
			(0.056)
Lack of capacity			0.724***
			(0.081)
Opinion about services in the area: Electricity supply	1.082**	1.083**	1.082**
	(0.034)	(0.034)	(0.034)
Opinion about services in the area: Bus services	1.086***	1.088***	1.071**
	(0.031)	(0.031)	(0.030)
Quality of government services provided by State Govnmt compared to 3 yrs ago	1.038	1.055	1.032
	(0.059)	(0.061)	(0.059)
Membership of religious group	1.192**	1.186**	1.176*
	(0.102)	(0.102)	(0.101)
Understanding of subsidy granted	0.944	0.956	0.951
	(0.084)	(0.085)	(0.085)
Big impact of 2016 fuel price increase (binary)	0.917	0.913	0.961
	(0.080)	(0.080)	(0.082)
Observations	12213	12185	12185

Robust standard errors clustered at the enumeration area in parentheses.
Region dummies are included in models.
We report the estimated coefficients and standard errors transformed to odds ratios.
Results for control variables in models are omitted.
**p < 0.10, ** p < 0.05, *** p < 0.01*

Table A15: Factors that influence support for subsidy reform: Logistic regression estimations results using a binary indicator for big impact of 2016 fuel price increase. Big impact of 2016 fuel price increase (binary) is coded as 1 if a response is either big (4) or very big impact (5) to the question, "Size of impact of 2016 fuel price increase." The results for control variables are omitted in the table.
